# Defender self-efficacy and moral disengagement on social support and bystander behaviors among primary school students: a multilevel moderated mediation model

**DOI:** 10.1186/s41155-023-00253-3

**Published:** 2023-04-28

**Authors:** Yuping Wu, Yanfang Zhou, Leishan Shi

**Affiliations:** 1 Teacher Education College, Lishui University, Lishui, Zhejiang Province People’s Republic of China; 2 Department of Psychology, Zhejiang Sci-tech University, Hangzhou, Zhejiang Province People’s Republic of China

**Keywords:** Bullying, Bystander behavior, Defender self-efficacy, Social support, Moral disengagement

## Abstract

This study examines the influence of social support on bystander behaviors, the mediating and moderating effects of moral disengagement and defender self-efficacy at the individual and class levels, and their cross-level interaction. A total of 1310 children in grades 4–6 completed our questionnaire survey at four-time points between October and December in 2021. The questionnaires include the Scale of Perceived Social Support (T1), Moral Disengagement Scale (T2), Defender Self-Efficacy Scale (T3), and Bullying Participant Behaviors Questionnaire (T4). The multilevel moderated mediating model results show that (1) social support negatively predicts reinforcer and outsider behavior and positively predicts defender behavior; (2) defender self-efficacy plays a mediating role between social support and defender behavior, moral disengagement plays a mediating role between social support and bystander behaviors, and defender self-efficacy and moral disengagement play a chain mediation role between social support and bystander behavior; (3a) class-level defender self-efficacy has a direct impact on defender behavior and moderates the relationship between individual defender self-efficacy and reinforcer behavior; and (3b) class-level moral disengagement has a direct impact on defender and outsider behavior and a cross-level moderated role between individual moral disengagement and reinforcer behavior. These results show that the individual and class level defender self-efficacy and moral disengagement can influence the bystander behavior of primary school students, which is of great significance for schools to develop anti-bullying moral education courses and design measures to improve students’ anti-bullying self-efficacy.

## Introduction

Bullying is commonly defined as intentional and repeated aggression directed at individuals who have fewer power advantages in certain interactions (Olweus, 2013). School bullying is a social group event that can cause severe psychological and social adjustment problems among children and adolescents (Olweus, 2013). In addition to bullies and victims, bystanders are also an important group in school bullying (Thornberg et al., 2017). Bystanders are any individuals who witness a bullying conflict (Pozzoli et al., 2012; Thornberg et al., 2017), regardless of their willingness (Balakrishnan, 2018). Three different bystander roles can emerge in bullying situations: reinforcers, defenders, and outsiders (Salmivalli et al., 1996; Thornberg et al., 2017). Defenders provide direct help or choose an indirect way of reporting to teachers or providing comfort to mitigate victimization. Reinforcers assist and participate in bullying, while outsiders take no action, allowing the bullying to develop or leaving the bullying scene, remaining passive (Stueve et al., 2006).

Increased defender behaviors reduce the occurrence of bullying, while increased reinforcer and outsider behaviors increase its likelihood (Polanin et al., 2012). Bystanders’ interaction with bullies or victims can mitigate the development of bullying incidents (Tsang et al., 2011). Therefore, exploring the factors and conditions related to bystander behavior is essential for understanding why some students act as defenders, reinforcers, or outsiders when witnessing school bullying. Previous research on school bullying has focused mainly on the binary relationship between bullies and victims; research on bystanders is still limited.

Social support can affect bystander behavior, making its influence mechanism worth exploring (Riffle & Demaray, 2021; Wood et al., 2017). Bystander behavior has group characteristics (Thornberg et al., 2017) and can be influenced by group factors (Salmivalli et al., 2011). The class context is an important factor when discussing bystander behaviors (Pozzoli et al., 2012; Salmivalli et al., 2011; Thornberg et al., 2017); however, the existing research on bystander behavior has focused mainly on individual factors such as empathy (Barchia & Bussey, 2011; Nickerson et al., 2008), moral disengagement (Thornberg & Jungert, 2013; Thornberg et al., 2015), self-efficacy (Barchia & Bussey, 2011; Pöyhönen et al., 2012; Thornberg & Jungert, 2013), and coping strategies (Pozzoli & Gini, 2012). Limited studies have examined contextual factors in class or school settings, such as collective efficacy (Barchia & Bussey, 2011), class norms (Salmivalli & Voeten, 2004), and moral disengagement at the classroom level (Gini et al., 2015; Pozzoli et al., 2012). Moreover, these studies only examine either individuals or groups (Jenkins & Nickerson, 2017), rarely investigating the influencing factors and interactions at both the individual and group levels (Pozzoli et al., 2012; Thornberg et al., 2017). It is necessary to identify the influencing factors and mechanisms at the individual level in the class context; thus, this study explores the influence of social support on bystander behavior in the classroom context.

### Social support and bystander behaviors

Social support refers to the emotional tools and knowledge provided to individuals by families, friends, and others (Thoits, 2011) and is typically regarded as a psychological resource. According to social support and nonsupport frameworks (Wood et al., 2017), individuals who feel enough social support are more inclined to participate in positive bystander behavior and offer victims help. In contrast, individuals who perceive inadequate effective social support are more likely to engage in passive bystander behavior. Defenders typically have more social support (Jenkins & Fredrick, 2017), whereas reinforcers and outsiders perceive less support from teachers, classmates, and friends (Riffle & Demaray, 2021; Wood et al., 2017). Social support from the network can promote defender behavior (Wood et al., 2017). Bystanders’ social support system, thus, has an important influence on their behavioral choices; however, research investigating how social support affects bystander behavior is still insufficient (Riffle & Demaray, 2021; Wood et al., 2017).

### Defender self-efficacy and moral disengagement at the individual level

Moral disengagement presents a potential path for the influence of social support on bystander behavior (Thornberg et al., 2017). Bandura (1999) proposed moral disengagement as an important cognitive concept to explain moral behavior, based on social cognitive theory. Moral disengagement refers to a self-regulated mechanism that underscores individuals’ cognitive tendencies to redefine their inhuman behaviors without any feelings of remorse or guilt. It can also justify behaviors to seem more reasonable and less harmful. Moral disengagement minimizes individuals’ responsibility and the consequences of behaviors, thus weakening their identification with the pain of victimization (Bandura, 1999). Moral disengagement includes eight mechanisms: moral justification, euphemistic language, advantageous comparison, displacement of responsibility, diffusion of responsibility, distorting consequences, attribution of blame, and dehumanization. In the context of school bullying, bystanders can usually recognize that bullying is immoral; however, individuals are less prone to be defenders to prevent bullying when they are on the sidelines.

According to social cognitive theory, individual behavior, environment, and cognition interact with each other. Negative environmental factors (i.e., lack of social support) can cause deviations in moral cognition and weaken the function of moral norms; therefore, individuals tend to use moral disengagement to compensate for cognition (Runions et al., 2019), which affects their choices (Bandura et al., 1996).

Research has shown that higher moral disengagement is related to more reinforcer and outsider behaviors and fewer defender behaviors (Sjögren et al., 2020). Higher moral disengagement is more likely to encourage bullying through cheering behaviors, while a lower level of moral disengagement makes individuals more inclined to protect victims through positive behaviors (Thornberg & Jungert, 2013). Social support can also inhibit moral disengagement (Shen et al., 2019). Reduced support from mothers and teachers is related to higher levels of moral disengagement among children (Campaert et al., 2017). In recent years, studies have introduced moral disengagement as an intermediary variable of social support, while individual behavior is considered to be a dominant variable (Stanger et al., 2018). However, it remains unclear whether moral disengagement is an intermediary mechanism for social support that influences bystander behavior in school bullying.

Defender self-efficacy is another potential mechanism for social support that influences bystander behavior. Based on social cognitive theory, moral behavior is relevant to moral disengagement and depends on individuals’ belief in their ability to act in adherence to moral standards. Self-efficacy is a belief in one’s ability to affect situations. Defender self-efficacy refers to the belief that individuals can intervene and protect victims successfully (Thornberg et al., 2017). However, intervening in school bullying is risky. Individuals who feel confident enough to successfully help victims out of their current predicament are more prone to act as defenders, while individuals who lack the confidence to implement protective behaviors exhibit restrained intervention (Thornberg & Jungert, 2013).

Defender self-efficacy has a stronger relationship with defender behaviors (Pöyhönen et al., 2012) and a weaker association with outsider behavior (Sjögren et al., 2020; Thornberg et al., 2017, 2020) in school bullying settings. Thus, individuals with higher defender self-efficacy are more willing to protect victims’ rights (Pöyhönen & Salmivalli, 2008). However, there are inconsistencies in the findings on defender self-efficacy and reinforcer behavior. Thornberg and Jungert (2013) found that defender self-efficacy is related to fewer reinforcer behaviors, while Pöyhönen et al. (2012) indicated that the relationship between defender self-efficacy and reinforcer behavior is not significant with the addition of additional variables. Therefore, the relationship between defender self-efficacy and bystander behavior requires further investigation (Thornberg & Jungert, 2013). Furthermore, verbal persuasion and alternative experiences are two important sources of self-efficacy. Thus, social support may affect individual defender self-efficacy (Bian et al., 2013). Individuals who do not believe that they have the ability to intervene in bullying require more support from a positive social support system to enhance their confidence and willingness to intervene (Wood et al., 2017).

Moral disengagement and defender self-efficacy may form a mechanism between social support and bystander behavior. Self-efficacy affects individuals’ behavior, willingness to make efforts, thinking processes, and emotional responses. In school bullying settings, individuals with low defender self-efficacy use moral disengagement to refuse to engage in protective actions, act as outsiders or reinforcers, and reduce their feelings of guilt and self-blame. Research has shown a negative correlation between emotional self-efficacy and moral disengagement (Ma & Jiao, 2019): Academic self-efficacy has a direct negative relationship with moral evasion and influences academic misconduct through the intermediary of moral disengagement (Fu & Wu, 2013), while interpersonal self-efficacy impacts cyberbullying through the partial mediation of moral disengagement (Liu et al., 2020).

According to Derr et al. (2020), defender self-efficacy and moral disengagement play separate mediating roles between personality growth mentality and defender behavior. However, the joint function of defender self-efficacy and moral disengagement between social support and bystander behavior remains unclear. Based on the above theory and previous empirical research, we propose that defender self-efficacy and moral disengagement have multiple mediating effects on social support and bystander behaviors in school bullying.

### Class-level defender self-efficacy and moral disengagement

In China, most schools are based on class-teaching systems. Students are generally concentrated in a fixed class to study together over a prolonged period with a consistent teacher and curriculum. Children are often closer to their classmates in fixed classes. The social ecological theory is important for understanding the bullying behavior of children and adolescents (Espelage & Swearer, 2010). The theory states that people and the environment in a microsystem directly interact with individuals and significantly influence the establishment, maintenance, and change of bystander behavior (Espelage & Swearer, 2010). The classroom is, thus, a critical microsystem that researchers should consider when discussing bystander behavior (Pozzoli et al., 2012; Salmivalli et al., 2011; Thornberg et al., 2017) as it is essential for understanding and solving school bullying (Gini et al., 2015; Salmivalli et al., 2011).

The big-fish-little-pond effect holds that pupils adopt the average ability of classmates as a frame of reference for comparison. Changes in a class’s average ability levels can impact individual self-concept, affecting students’ performance (Marsh et al., 2008). Therefore, this study analyzes the influence of defender self-efficacy and moral disengagement on bystander behavior in the class context. Changes in the class environment can also adjust children’s behavior, as they may refer to others’ bystander behavior. For example, when other classmates exhibit high levels of moral disengagement, the whole class’s level will be high, creating an atmosphere of moral disengagement that may affect the moral disengagement of each pupil. Similarly, high levels of defender self-efficacy among others can increase the average self-efficacy level of the whole class. A positive environment is then formed, in which most students believe in their ability to help victims, thus influencing individual defender self-efficacy and bystander behavior in the class. Consequently, it is pertinent to investigate class-level factors to further understand the influence of social support on individual bystander behavior based on individual-level variables.

Morality is learned and cultivated through social interactions, and moral behavior is influenced by both individuals and society (Bandura, 2002). Thus, morality can occur at both the individual and collective levels. The transfer of responsibility between groups also promotes human behavior (Gini et al., 2015). Class-level moral disengagement has been operationalized by aggregating individual moral disengagement (Thornberg et al., 2017). Class moral disengagement is a group characteristic describing the potential to affect group members’ cognition and behavior. Moral disengagement in a class is related to reinforcer behavior (Pozzoli et al., 2012). Outsiders are prevalent in classes with a high degree of moral disengagement, while defender behavior is common in classes with low levels of moral disengagement (Gini et al., 2015). Thornberg et al. (2017) also examined the interaction between individual- and class-level moral disengagement and found that the relationship between individual moral disengagement and outsider behavior was significantly influenced by moral disengagement. However, whether the relationship between individual moral disengagement and other bystander behaviors differs across various classes remains unknown. In the study, similar to class moral disengagement, individual defender self-efficacy was aggregated at the class level to form class-average defender self-efficacy as the group characteristic. While several studies have discussed the moral disengagement and self-efficacy of defenders at the individual level, it remains unclear whether these two class environmental factors, aggregated by individuals, can, in turn, influence individual bystander behavior.

### The present study

**Fig. 1:**
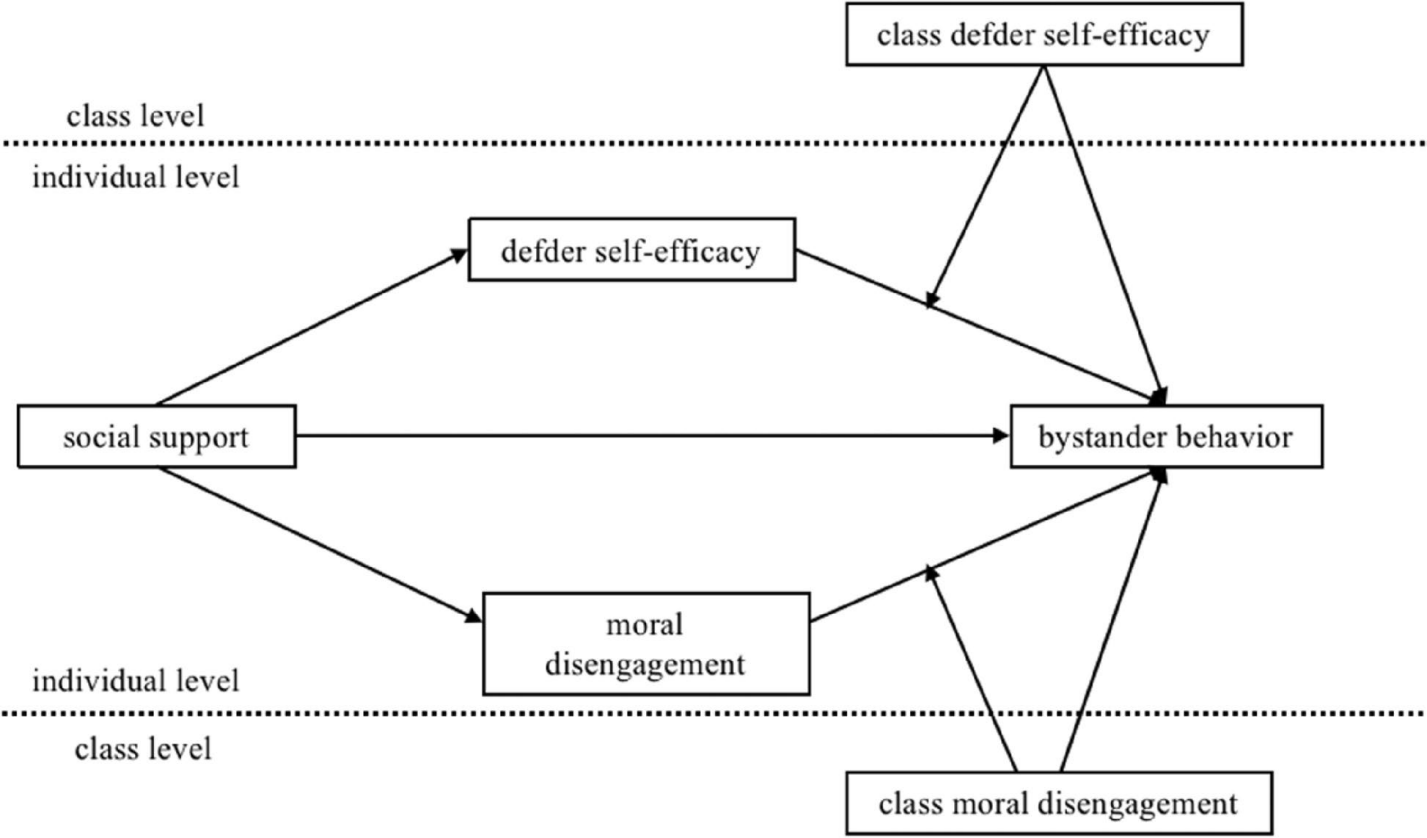
Conceptual multilevel moderated mediation model

Based on the social support and nonsupport frameworks and social cognitive theory, this study investigates the relationship between social support and bystander behavior and proposes a multilevel moderated mediation model using age, gender, and class size as control variables (Fig. 1).

This study hypothesized that (1) social support positively predicts defender behavior and negatively predicts reinforcer and outsider behavior, (2) defender self-efficacy and moral disengagement mediate the relationship between social support and bystander behaviors, (3a) class-level defender self-efficacy has a direct effect on bystander behaviors and a cross-level moderating effect on the mediating pathway (i.e., the second half) in which social support affects bystander behavior via defender self-efficacy, and (3b) class-level moral disengagement has a direct effect on bystander behaviors and a cross-level moderating effect on the mediating pathway (i.e., the second half), in which social support affects bystander behavior via moral disengagement.

## Methods

### Participants

The sample comprised 1310 pupils (aged 8 to 14 years; mean = 10.97, standard deviation (SD) = 0.98) from 61 classes in three primary schools in Lishui, Zhejiang. The participants were 616 boys (47.00%), 685 girls (52.30%) and 9 individuals (0.70%) with missing gender data. Among them, 302 (23.10%) were in grade 4, 416 (31.80%) were in grade 5, and 592 (45.20%) were in grade 6. The original sample included 1326 learners; however, 16 pupils did not complete all four measurements during the measurement process and were, thus, excluded from the analysis. The 61 classes had 30 to 50 students each, with an average of 42.13 ± 3.48 per class and an average of 21.47 ± 7.26 participants in each class. In total, there were 18, 20, and 23 classes in grades 4, 5, and 6, respectively.

### Procedure

Children participated in a survey four times from October to December 2021. Social support was measured at T1, moral disengagement was measured at T2 (approximately 1 week after T1), defender self-efficacy was measured at T3 (approximately 2 weeks after T2), and bystander behavior was measured at T4 (approximately 4 weeks after T3). This procedure controlled deviation from common methods. Collective measurements were used. All the data were collected during lunch breaks in the classroom at each time period. Teachers were not present during data collection, which was conducted by psychology graduate students. The students explained the test procedure, guaranteed confidentiality for all information shared, and helped participants requiring assistance. Questionnaires were collected immediately after completion.

### Measures

#### Socio-demographic scale

Participants completed a socio-demographic scale containing several questions about their name (i.e., “What is your name?”), gender (0 = girl, 1 = boy), and age (i.e., “How old are you?” followed by, ‘I'm …… years and …… months old’), and class (i.e., “What is your class?” followed by, “I’m in Grade …… and class……”).

#### Social support at time 1

Social support was measured using the 12-item Multidimensional Scale of Perceived Social Support (MSPSS; Zimet et al., 1988). Following Yan and Zheng (2006), “leaders, relatives, and colleagues” was changed to “parents, friends, and teachers.” Responses were scored using a 7-point Likert scale (1 = *strongly disagree* to 7 = *strongly agree*). The average score of the items indicated the degree of social support that participants perceived. Cronbach’s alpha was 0.90 in the current study.

#### Moral disengagement at time 2

The Chinese version of the Moral Disengagement Scale (CMDS) was developed based on the Moral Disengagement Scale (Bandura et al., 1996). The CMDS comprises 26 items assessing moral disengagement, such as “it is alright to fight to protect friends,” “it is okay to insult a classmate because beating them is worse,” and “it is unfair to blame a child who has only a small part in the harm caused by a group.” Responses are scored on a 5-point scale (1 = *strongly disagree* to 5 = strongly agree). All item responses were averaged, with higher scores indicating higher moral disengagement. In this study, Cronbach’s alpha was 0.92.

#### Defender self-efficacy scale at time 3

The five-item Defender Self-Efficacy Scale was used (Thornberg et al., 2017). Participants were asked to estimate the extent to which the following statements were true: “I feel that I’m very good at…,” followed by five items, “…telling off/standing up to students who are mean toward another student,” “…helping students who are bullied,” “…stopping bullying,” “…telling students who are bullying someone to stop doing that,” and “…ensuring a group stops making up stories/lying about another student.” The response options for each item were rated on a 7-point scale (1 = *disagree* to 7 = *agree*). Thereafter, the average of these five items was computed for each student (Cronbach’s α = 0.90).

#### The bullying participant behaviors questionnaire at time 4

Bystander behavior was measured using the Bullying Participant Behaviors Questionnaire (BPBQ; Demaray et al., 2016). Three independent dimensions of the measurement were adopted, and each subscale has 10 items, rated on a 5-point Likert scale. The participants were informed of the definition of bullying and asked to rate questions based on their experiences in the past 30 days. The assistant subscale assesses the frequency of participating in or encouraging bullying (e.g., “When someone was making fun of another student, I joined in,” “I have made fun of someone when they were pushed, punched, or slapped,” “When someone else broke something that belonged to another student, I stopped watching” (Cronbach’s α = 0.96)). The defender subscale assesses the frequency of standing up for victims (e.g., “I encouraged someone to tell an adult after they were picked on,” “I defended someone who was being pushed, punched, or slapped” (Cronbach’s α = 0.90)). The outsider subscale assesses the frequency of ignoring bullying (e.g., “I ignored it when I saw someone making fun of another student,” “I pretended not to notice a situation that purposely left someone out” (Cronbach’s α = 0.95)).

### Data analysis

The maximum likelihood method was used to address missing data. First, SPSS 26.0 was used for the initial descriptive analyses and bivariate correlations. Second, structural equation modeling (SEM) in Mplus 8.3 was used while bootstrapping; 1000 samples were applied to test the significance of the mediated effects and produce bias-corrected percentile confidence intervals. Social support at T1 was the independent variable, while defender self-efficacy at T2 and moral disengagement at T3 were the intermediary variables. Reinforcer behavior, defender behavior, and outsider behavior at T4 were the dependent variables. Structural model fit was analyzed using the Tucker-Lewis index (TLI), comparative fit index (CFI), and root mean square error of approximation (RMSEA), which was applied with a 90% confidence interval (CI; Byrne, 2001). For the CFI and TLI, values greater than 0.90 were considered acceptable, and values greater than 0.95 were considered a good fit for the data (Byrne, 2001). For RMSEA, values less than 0.06 indicated a good fit, while values between 0.06 and 0.10 were considered adequate (Byrne, 2001). If the 95% CI for the indirect effect estimate did not include zero, the indirect effect was found to be statistically significant at the 0.05 level. The size of the mediating effect was also calculated.

Third, two class-level factors, moral disengagement and defender self-efficacy, were added to the mediating model. Multilevel structural equation modeling (MSEM) in Mplus 8.3 was used to test the significance of the cross-level moderating effect. The class moral disengagement and defender self-efficacy were measured using two paths: individual moral disengagement-bystander behavior and defender self-efficacy-bystander behavior. The intraclass correlation coefficient (ICC) of the major variables was then calculated. The ICC(1) of defender self-efficacy and moral disengagement were 0.032 and 0.102, respectively; the mean RWG was 0.95, the median was 0.94, and the ICC(2) was 0.85. Thus, data aggregation in this study was feasible.

## Results

### Descriptive statistics and correlations

The descriptive statistics and bivariate correlations of the study variables and covariates are presented in Table 1. At the individual level, social support was negatively associated with moral disengagement, reinforcer behavior, and outsider behavior but positively associated with defender self-efficacy and defender behavior. Moral disengagement was positively associated with reinforcer behavior and outsider behavior and negatively associated with defender self-efficacy and defender behavior. Reinforcer behavior was positively associated with outsider behavior and negatively associated with defender self-efficacy. At the class level, moral disengagement was negatively associated with defender self-efficacy.

**Table 1:** Descriptive statistics and correlations among the variables

	M	SD	1	2	3	4	5	6	7	8
Individual level										
Gender	—	—	—							
Age	10.97	0.98	− 0.05	1						
Social support	5.93	1.05	− 0.02	0.14^***^	1					
Moral disengagement	1.67	0.47	0.01	− 0.08^**^	− 0.29^***^	1				
Defender self-efficacy	5.46	1.37	− 0.46	0.07^**^	0.39^***^	− 0.28^***^	1			
Reinforcer behavior	0.14	0.39	− 0.05	0.06^*^	− 0.14^***^	0.22^***^	− 0.11^***^	1		
Defender behavior	3.12	0.99	0.02	0.03	0.28^***^	− 0.23^***^	0.37^***^	− 0.11^***^	1	
Outsider behavior	1.51	0.99	− 0.06^*^	0.06^**^	− 0.16^***^	0.19^***^	− 0.10^**^	0.31^***^	− 0.03	1
Class level										
Grade	—	—	—							
Class size	42.13	3.48	− 0.06^*^	1						
Class moral disengagement	1.67	0.18	− 0.33^***^	0.00	1					
Class defender self-efficacy	5.46	0.37	0.24^***^	− 0.13^***^	− 0.55^***^	1				

^*^p < 0.05, ^**^p < 0.01, ^***^p < *0.001*

The Harman single-factor method was used to test the common method biases, and the explanation percentage of the first-factor variance was 5.07%. This value was less than the critical standard of 40%, indicating that the common method variation of the study data was not severe.

### Reinforcer behavior

**Fig. 2:**
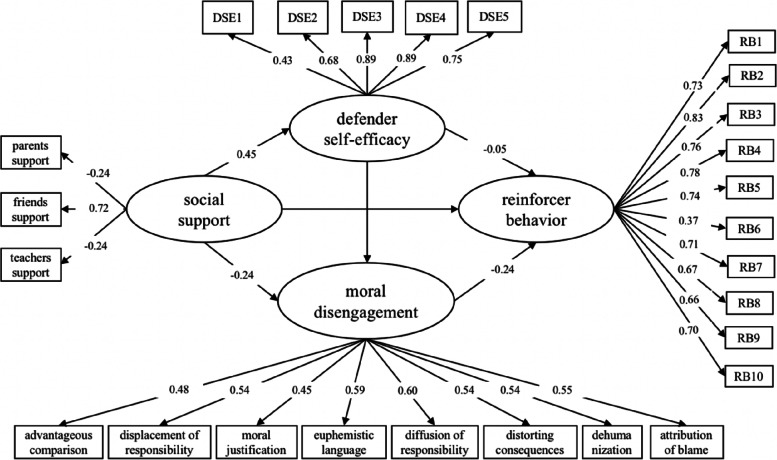
The mediating effect of defender self-efficacy and moral disengagement in the relationship between social support and reinforcer behavior

The mediation model consisted of social support, moral disengagement, defender self-efficacy, and reinforcer behavior. A model that considered all paths revealed an acceptable fit to the data (RMSEA = 0.049, SRMA = 0.049, CFI = 0.918, TLI = 0.910). As shown in Fig. 2, the relationship between social support and reinforcer behavior was not significant after controlling for age and gender. However, social support positively predicted defender self-efficacy ($\beta$ = 0.45, SE = 0.03, *p* < 0.001), which was negatively correlated with moral disengagement (*β* =  − 0.25, SE = 0.04, *p* < 0.001). Furthermore, defender self-efficacy was negatively correlated with moral disengagement (*β* =  − 0.26, SE = 0.04, *p* < 0.001).

The indirect effect of social support on reinforcer behavior through defender self-efficacy was not statistically significant (Table 2). However, the indirect effect of social support on reinforcer behavior through moral disengagement was statistically significant ($\beta$ = − 0.05, SE = 0.01, *p* = 0.001). Furthermore, the indirect effect of social support on moral disengagement through defender self-efficacy, which in turn influences reinforcer behavior, was also significant ($\beta$ = − 0.02, SE = 0.01, *p* = 0.003). Thus, the total size of the mediating effect of self-efficacy and moral disengagement on defender behavior was 82.91% (− 0.097/ − 0.117).

**Table 2:** Summary of the direct and indirect effects

Direct and indirect effects	Estimate	SE	95%CI	*p*
Social support → defender self-efficacy → reinforcer behavior	− 0.02	0.02	[− 0.056, 0.020]	0.316
Social support → moral disengagement → reinforcer behavior	− 0.05	0.02	[− 0.085, − 0.025]	0.001
Social support → defender self-efficacy → moral disengagement → reinforcer behavior	− 0.02	0.01	[− 0.043, − 0.011]	0.003

### Defender behavior

**Fig. 3:**
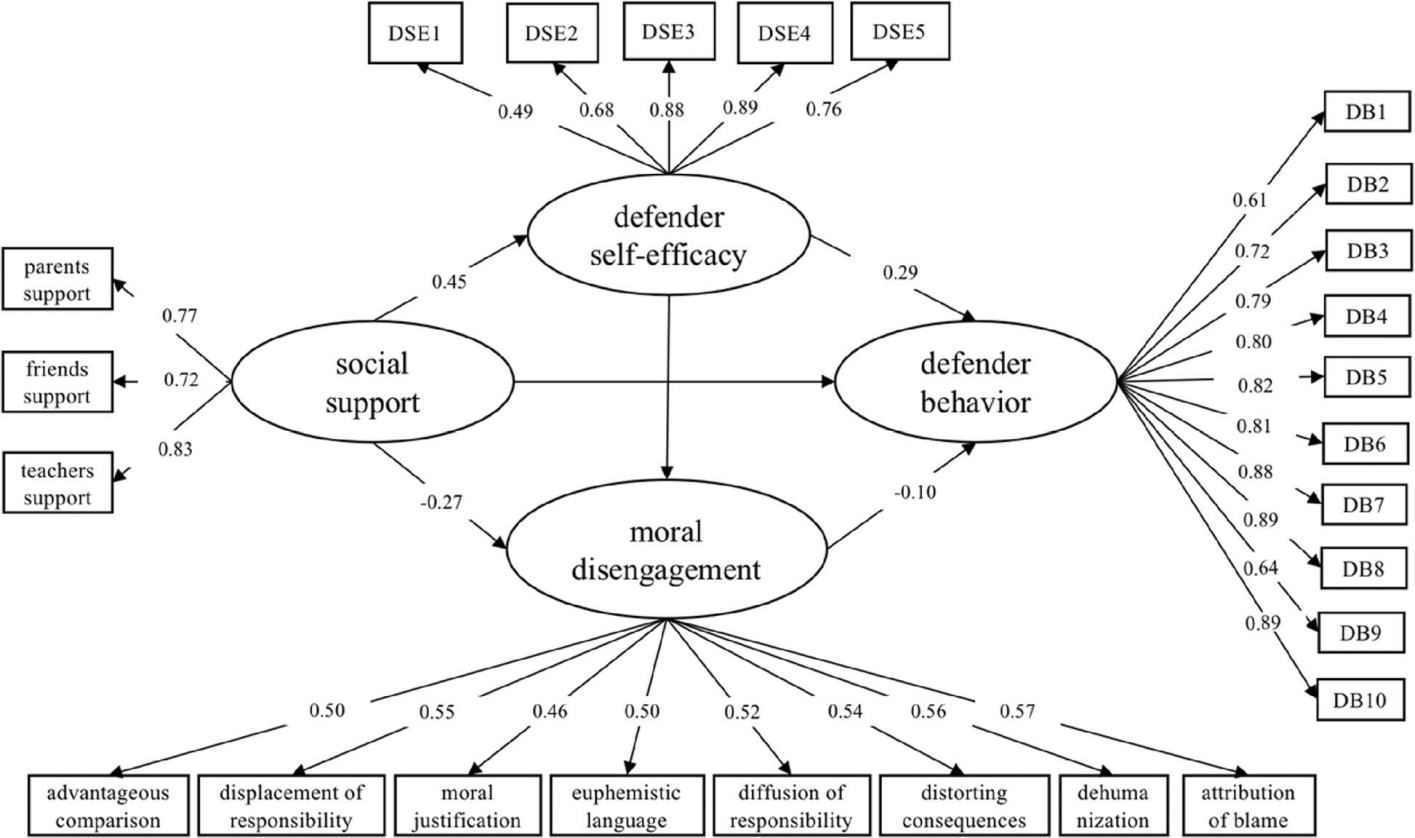
The mediating effect of defender self-efficacy and moral disengagement in the relationship between social support and defender behavior

The mediation model consisted of social support, moral disengagement, defender self-efficacy, and defender behavior. A model considering all paths revealed an acceptable fit to the data (RMSEA = 0.060, SRMA = 0.061, CFI = 0.916, TLI = 0.907). After controlling for age and gender, social support significantly positively predicted defender behavior ($\beta$ = 0.14, SE = 0.04, *p* < 0.001) and defender self-efficacy ($\beta$ =0.45, SE = 0.03, *p* < 0.001), which was negatively correlated with moral disengagement ($\beta$ = − 0.27, SE = 0.05, *p* < 0.001) (Fig. 3). Defender self-efficacy was positively correlated with defender behavior ($\beta$ = 0.29, SE = 0.03, *p* < 0.001) and negatively correlated with moral disengagement ($\beta$ = − 0.26, SE = 0.04, *p* < 0.001). In contrast, moral disengagement was negatively correlated with defender behavior ($\beta$ = − 0.10, SE = 0.04, *p* = 0.012).

The indirect effect of social support on defender behavior through defender self-efficacy was statistically significant ($\beta$ = 0.13, SE = 0.02, *p* = 0.012) (Table 3), as was the indirect effect of social support on reinforcer behavior through moral disengagement ($\beta$ = 0.03, SE = 0.01, *p* = 0.026). Furthermore, the indirect effect of social support on moral disengagement through defender self-efficacy, which in turn influences defender behavior, was also significant ($\beta$ = 0.01, SE = 0.01, *p* < 0.001). Thus, the total size of the mediating effect of self-efficacy and moral disengagement on defender behavior was 53.13% (0.17/0.32).

**Table 3:** Summary of the direct and indirect effects

Direct and indirect effects	Estimate	SE	95%CI	*p*
Social support → defender self-efficacy → defender behavior	0.13	0.02	0.003, 0.068	0.012
Social support → moral disengagement → defender behavior	0.03	0.01	0.068, 0.126	0.026
Social support → defender self-efficacy → moral disengagement → defender behavior	0.01	0.01	0.015, 0.037	0.000

### Outsider behavior

**Fig. 4:**
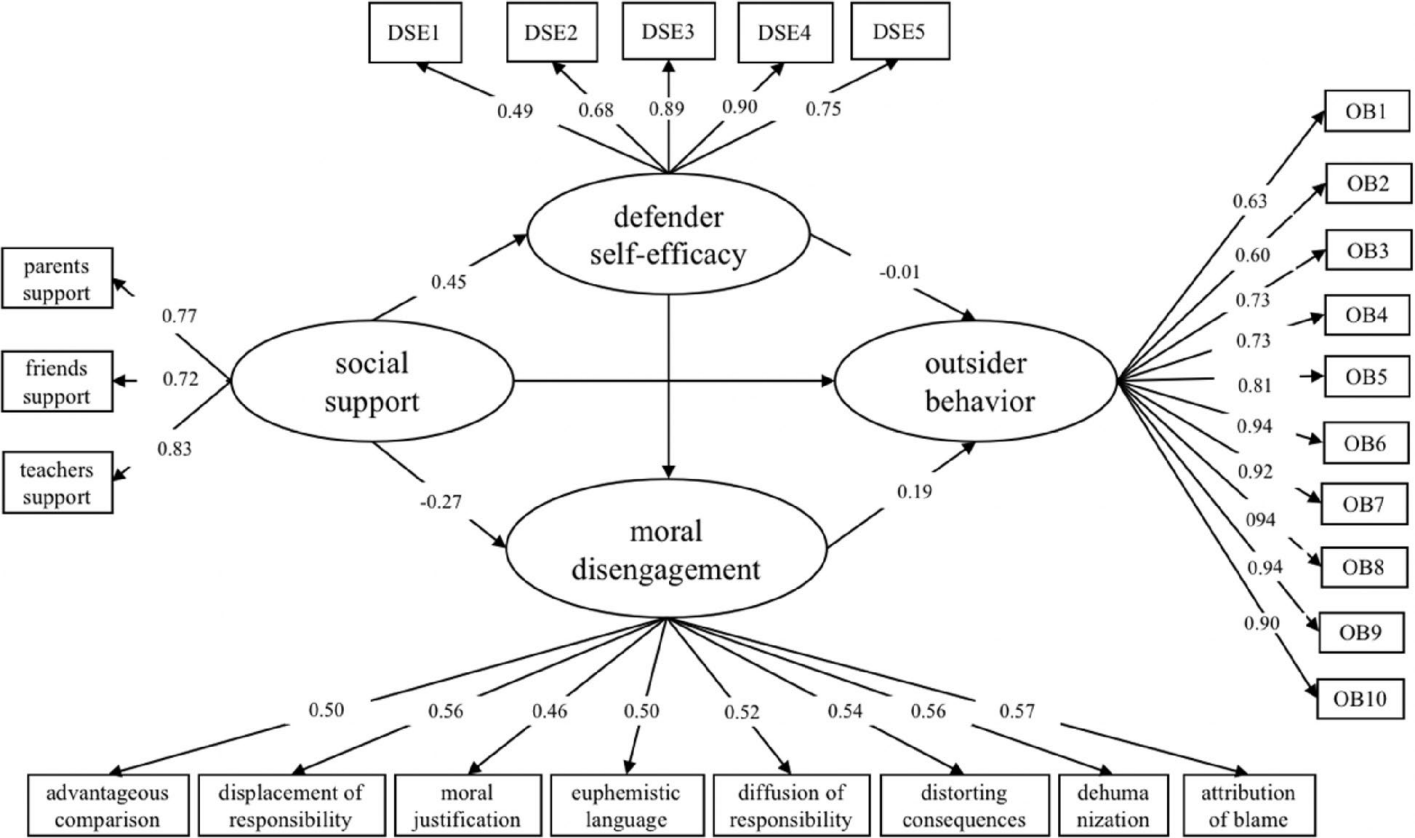
The mediating effect of defender self-efficacy and moral disengagement in the relationship between social support and outsider behavior

The mediation model consisted of social support, moral disengagement, defender self-efficacy, and outsider behavior. A model that considered all paths revealed an acceptable fit to the data (RMSEA = 0.051, SRMA = 0.040, CFI = 0.943, TLI = 0.937). Figure 4 indicates that the relationship between social support and outsider behavior was not significant after controlling for age and gender. However, social support positively predicted defender self-efficacy ($\beta$ = 0.45, SE = 0.032, *p* < 0.001), which was negatively correlated with moral disengagement ($\beta$ = − 0.27, SE = 0.049, *p* < 0.001). Defender self-efficacy was not significantly correlated with moral disengagement but was negatively correlated with moral disengagement ($\beta$ = 0.19, SE = 0.060, *p* = 0.001). Moral disengagement was negatively correlated with outsider behavior ($\beta$ = − 0.26, SE = 0.044, *p* < 0.001).

The results in Table 4 show that the indirect effect of social support on outsider behavior through defender self-efficacy was not significant. However, the indirect effect of social support on outsider behavior through moral disengagement was statistically significant ($\beta$ = − 0.05, SE = 0.01, *p* = 0.026). Furthermore, the indirect effect of social support on moral disengagement through defender self-efficacy, which in turn influences defender behavior, was also significant ($\beta$ = 0.01, SE = 0.02, *p* = 0.004). Thus, the total size of the mediating effect of self-efficacy and moral disengagement on outsider behavior was 44.44% (− 0.08/ − 0.18).

**Table 4:** Summary of the direct and indirect effects

Direct and indirect effects	Estimate	SE	95%CI	*p*
Social support → defender self-efficacy → outsider behavior	− 0.01	0.02	− 0.042, 0.039	0.953
Social support → moral disengagement → outsider behavior	− 0.05	0.02	− 0.095, − 0.021	0.004
Social support → defender self-efficacy → moral disengagement → outsider behavior	− 0.02	0.01	− 0.043, − 0.009	0.010

### Moderated mediation effect

To test for completely multi-layered moderated mediating effects, we included social support, individual and class moral disengagement, individual and class defender self-efficacy, and bystander behavior in a multilevel structural equation. Following Bauer et al. (2006) and Hayes (2013), we estimated the mediating effects when the moderating variable was high with low levels of class defender self-efficacy and class moral disengagement (at mean + 1SD and − 1SD). A significant difference between high (+ 1 SD) and low (− 1 SD) for the class-level variable indicates a moderating mediating effect at multiple levels.

### Reinforcer behavior

The indirect effect of social support on reinforcer behavior was not significant with high class-level defender self-efficacy with a 95% CI (CI = [− 0.052, 0.007]; zero included). However, this indirect effect was not significant with low class-level defender self-efficacy (CI = [− 0.024, 0.049]; zero included). The indirect effect was significant for the class-level defender self-efficacy difference (CI = [− 0.070, − 0.001]). Therefore, a multilevel moderated mediation effect was established. Class-level defender self-efficacy moderated the mediation path based on social support, which influenced reinforcer behavior through individual defender self-efficacy.

In the same model, the indirect effect of social support on reinforcer behavior was examined when the moderation variable was high with low levels of class defender self-efficacy and class moral disengagement (at mean + 1SD and − 1SD). The influence of high-class moral disengagement level on the indirect path between social support and reinforcement behavior was significant; the 95% CI was zero excluded (CI = [− 0.082, − 0.012]). However, it was not significant under low-class moral disengagement level with a 95% CI that was zero included (CI = [− 0.016, 0.012]). The indirect effect was significant at the class level of moral disengagement (CI = [− 0.070, − 0.001]). A multilevel moderated mediation effect was, thus, established. Class-level moral disengagement moderated the mediation path based on social support, affecting reinforcer behavior through individual moral disengagement.

Compared with the class environment with low-defender self-efficacy, when individuals were in a class with low-defender self-efficacy, the negative relationship between individual defender self-efficacy and reinforcer behavior was stronger. In addition, we found that class-level defender self-efficacy and moral disengagement have no significant direct effect on defender behavior.

### Defender behavior

The indirect effect of social support on defender behavior was significant with high-class level defender self-efficacy, and the 95% CI was zero excluded (CI = [0.096, 0.156]). This indirect effect was also significant with low class-level defender self-efficacy (CI = [0.058, 0.125]; zero included). However, it was not significant at the class level of the defender self-efficacy difference (CI = [− 0.006, 0.075]), meaning a multilevel moderated mediation effect was not observed: class-level defender self-efficacy did not moderate the mediation path through which social support influenced defender behavior through individual defender self-efficacy.

In the same model, the indirect effect of social support on defender behavior was analyzed when the moderation variable was high with low levels of moral disengagement (at mean + 1SD and − 1SD). The influence of high levels of class moral disengagement on the indirect path between social support and defender behavior was not significant; 95% CI was zero included (CI = [− 0.004, 0.031]). Similarly, it was not significant under low levels of class moral disengagement (CI = [− 0.002, 0.031]). The indirect effect was also not significant at the class level of moral disengagement difference (CI = [− 0.025, 0.023]). A multilevel moderated mediation effect was not observed; thus, class-level moral disengagement did not moderate the mediation path based on social support affecting defender behavior through individual moral disengagement. However, defender self-efficacy had a significant positive and direct effect on defender behavior (*b* = 0.338, SE = 0.156, *p* = 0.030), while moral disengagement had a significant negative and direct effect on defender behavior (*b* =  − 0.290, SE = 0.123, *p* = 0.018).

### Outsider behavior

The indirect effect of social support on outsider behavior was significant with high class-level defender self-efficacy with a 95% CI that was zero included (CI = [− 0.047, 0.050]). However, the indirect effect was not significant with low class-level defender self-efficacy (CI = [− 0.075, 0.051]; zero included) nor was it significant at the class level of the defender self-efficacy difference (CI = [− 0.088, 0.113]). A multilevel moderated mediation effect was not observed; thus, class-level defender self-efficacy did not moderate the mediation path by which social support influences outsider behavior through individual defender self-efficacy.

In the same model, the indirect effect of social support on outsider behavior was examined when the moderation variable was high with low levels of moral disengagement (at mean + 1SD and − 1SD). The influence of high levels of class moral disengagement on the indirect path between social support and defender behavior was significant; the 95% CI was zero excluded (CI = [− 0.055, − 0.007]). In contrast, it was not significant at low levels of class moral disengagement (CI = [− 0.027, 0.003]). The indirect effect was also not significant at the class level of moral disengagement difference (CI = [− 0.027, 0.003]). Therefore, a multilevel moderated mediation effect was not observed. Moreover, class-level moral disengagement did not moderate the mediation path through which social support affects outsider behavior through individual moral disengagement. Class moral disengagement had a significant positive and direct effect on outsider behavior (*b* = 0.367, SE = 0.107, *p* = 0.001); however, class defender self-efficacy had no significant direct effect on outsider behavior.

## Discussion

Based on the social support and nonsupport frameworks and social cognitive theory, this study explored the mediation mechanism of defender self-efficacy and moral disengagement between social support and bystander behavior using the mediation effect model at the individual level. We then used a multilevel structural equation to examine the mediation and direct effect of class-level defender self-efficacy and moral disengagement on the individual-level indirect path. We found that defender self-efficacy and individuals’ moral disengagement play a mediating role between social support and bystander behavior, and class-level defender self-efficacy and moral disengagement moderate the mediation effect.

This study found that social support can negatively predict reinforcer behavior and outsider behavior and positively predict defender behavior, which is consistent with existing research (Jenkins & Fredrick, 2017; Riffle & Demaray, 2021; Wood et al., 2017) and verified H1. As a vital psychological resource, social support can reduce bystanders’ perceptions of danger when school bullying occurs, improve their ability and confidence to cope with stressful events, and help them take positive protective actions. Conversely, if social support is lacking, bystanders will consider bullying intervention beyond their ability and will engage in passive bystander behaviors such as outsider or reinforcer behaviors.

Defender self-efficacy played a mediated role between social support and defender behavior but had no effect between social support and reinforcer and outsider behavior. Consistent with previous studies (Pöyhönen et al., 2012; Thornberg & Jungert, 2013), individuals with good social support exhibited higher levels of defender self-efficacy, which can significantly predict increased defender behavior. Similar to existing studies, our findings indicate that defender self-efficacy has no mediating effect on the relationship between social support, reinforcer behavior, and outsider behavior (Pöyhönen et al., 2012). Social support can promote defender self-efficacy; however, the inclusion of additional variables yields an insignificant influence of defender self-efficacy on reinforcer and outsider behaviors. Thus, an individual’s self-efficacy is not sufficient to guarantee the realization of the expected behavior (Gini et al., 2015). Furthermore, the direct influence of defender self-efficacy on bystander behaviors may focus primarily on individuals who intend to act as defenders. Moreover, influence underscores that several other mechanisms may exist between defender self-efficacy and reinforcer and outsider behaviors. To a certain extent, our findings verify and supplement the conception of the relationship between social support, self-efficacy, and bystander behaviors proposed by Wood et al. (2017).

Moral disengagement mediated social support and bystander behavior, consistent with the existing research (Liu & Liu, 2020). Moral disengagement can, thus, improve the understanding of the influence of social support on bystander behavior. Furthermore, social support can also reduce the level of moral disengagement. Low moral disengagement is related to fewer reinforcers and outsider behaviors and more defender behaviors.

High defender self-efficacy is related to lower moral disengagement. Defender self-efficacy and moral disengagement play a chain-mediating role between social support and bystander behaviors, supporting this study’s hypothesis. Furthermore, the findings show that social support can reduce the level of moral disengagement by improving defender self-efficacy and increasing defender behavior while reducing reinforcer and outsider behavior. Self-efficacy directly affects an individual’s behavior, willingness to act, thinking process, and emotional response. Individuals who do not believe that they are able to successfully protect victims of school bullying are prone to retreat and escape. They also understand that bullying is immoral and, thus, may find excuses through moral disengagement mechanisms to feel justified in their refusal to be defenders and show more reinforcer and outsider behaviors. Therefore, the results of the individual-level mediation effect model support H2.

The class with higher defender self-efficacy displayed more defender behavior, which is consistent with existing research results (Barchia & Bussey, 2011; Sjögren et al., 2020). We examined the interaction between individual and class variables to determine the influencing mechanism of social support on bystander behaviors. Class-level defender self-efficacy strengthens the relationship between individual defender self-efficacy and reinforcer behavior, which is consistent with Thornberg et al. (2020). However, we did not find a significant cross-level interaction of defender self-efficacy with defender behavior and outsider behavior, only affecting defender behavior. This result is consistent with those of Sjögren et al. (2020). If other students in a child’s class have a high level of defender self-efficacy, their belief in protecting victims may be stimulated. Furthermore, a child in a class in which pupils have a low sense of defender self-efficacy will have a limited belief in their ability to protect victims. Therefore, compared with the class with low defender self-efficacy, individuals in the class with high defender self-efficacy showed less reinforcer behavior. Children who exhibit low defender self-efficacy do not believe that they can protect the victim. If the surrounding pupils also think that they cannot mitigate victimization, then the individual will be influenced by other students in the class, further limiting individual belief. Hence, it is easier to amplify the threat from the bully and become a reinforcer.

Consistent with existing research, the class with serious moral disengagement showed less defender behavior and more outsider behavior (Gini et al., 2015). A class with an environment in which people generally refuse to take responsibility will blame bullying on victims instead of punishing bullies. Consequently, the members of this class will think that they do not need to be responsible for the outcomes caused by their actions and are more prone to refuse to act as defenders, catering to other people’s attitudes or receiving pressure from the group, demonstrating more outsider behaviors.

Class-level moral disengagement also enhances the relationship between individual-level moral disengagement and reinforcer behavior. Thus, students with higher moral disengagement are more likely to become reinforcers if they are in a class with high moral disengagement. However, class moral disengagement had no significant cross-level moderating effect on the relationship between individual moral disengagement and defender and outsider behavior, which is consistent with the results of Gini et al. (2015). This may be because students with high moral disengagement are better at using these mechanisms to find excuses for their negative bystander behaviors. If their class generally tends to absolve their responsibilities as bystanders, their cognition will be further strengthened. Thus, these individuals are more likely to agree with the immoral behaviors of bullies and could further exhibit reinforcer behaviors. Therefore, the multilevel model supports H3.

Overall, our results support Wood et al.’s (2017) social support and nonsupport frameworks and are consistent with social cognitive theory. Social support can influence individual bystander behavior through defender self-efficacy and moral disengagement. Further, the mediation mechanism of social support on bystander behavior at the individual level is influenced by class factors; thus, the mechanism at the individual level was examined and the cross-level interaction was explored in accordance with cognitive society theory.

This study has two limitations that should be addressed in future research. First, although variable data were collected at four time periods, the research constitutes a cross-sectional study, which precludes drawing causal conclusions. The causal relationships between social support, moral disengagement, defender self-efficacy, and bystander behavior should be analyzed further through longitudinal research. Second, the scales used in this study were all self-reported; thus, a social praise effect may exist. Future research could use more methods, such as peer nomination, to collect data to measure bystander behavior.

This study’s findings have several implications for bystander behavior. First, establishing a good social support system can effectively increase bystanders’ positive interventions. Whether the influence is direct or indirect, good social support is conducive to positive behavior. Therefore, it is necessary to create good behavioral environments for children. Second, enhancing bystanders’ defender self-efficacy and reducing their moral disengagement can prevent individuals from contributing to or ignoring school bullying, enhancing their confidence to fight against it. Finally, the study’s multilevel moderated mediation model shows that lower defender self-efficacy and higher moral disengagement increase reinforcer behavior. Thus, increased focus should be placed on the interaction of “individual × class,” instead of merely focusing on one aspect of an individual or class to find the most effective way to promote positive bystander behaviors and reduce school bullying.

## Conclusion

This study explored the influence of social support on bystander behavior. A high level of social support promotes defender behavior by improving individual defender self-efficacy. It also increases defender behavior and reduces reinforcer and outsider behavior by restraining individual moral disengagement. Moreover, high-level social support can reduce moral disengagement by improving defender self-efficacy to help individuals produce more defender behaviors and less reinforcer and outsider behaviors. These results are consistent with social cognitive theory and complement the social support and nonsupport frameworks. In addition, classes with high defender self-efficacy were more likely to display defender behaviors and exhibit fewer reinforcer behaviors by enhancing personal defender self-efficacy. Furthermore, a class with generally high levels of moral disengagement may produce fewer defender behaviors and more outsider behaviors. Thus, as opposed to classes with low moral disengagement, social support in classes with high-level moral disengagement leads to fewer reinforcer behaviors by reducing moral disengagement.

## Data Availability

The data that support the findings of this study are available from the corresponding author, Leishan Shi, upon reasonable request.
